# Renal myelolipoma: a rare extra-adrenal tumor in a rare site: a case report and review of the literature

**DOI:** 10.1186/1752-1947-7-92

**Published:** 2013-04-04

**Authors:** Merieme Ghaouti, Kaoutar Znati, Ahmed Jahid, Fouad Zouaidia, Zakiya Bernoussi, Najat Mahassini

**Affiliations:** 1 Department of Pathology, Ibn Sina University Hospital, Rabat, Morocco; 2 Hopital Ibn Sina, Avenue Abderrahim Bouabid, Rabat, 11100, Morocco

**Keywords:** Extra-adrenal myelolipoma, Myelolipoma, Renal myelolipoma, Retroperitoneal tumors

## Abstract

**Introduction:**

Myelolipomas are uncommon, benign tumors composed of mature adipose tissue and hematopoietic elements. They mostly occur in the adrenal glands, but extra-adrenal myelolipomas have also been reported in other locations such as the presacral region, retroperitoneum, pelvis and mediastinum. Here, we present a case of an extra-adrenal myelolipoma in a rare site: the renal parenchyma. To the best of our knowledge, it is only the third case reported in this unusual location.

**Case presentation:**

We report a case of primary myelolipoma occurring in the kidney of a 55-year-old Moroccan man. We describe the radiological and clinicopathologic features of this unusual tumor with a review of the literature, and we discuss differential diagnosis of retroperitoneal myelolipomas.

**Conclusion:**

This case is noteworthy because the tumor site was unusual. Although renal myelolipoma is rare, it should be considered in the differential diagnosis of lesions in this site.

## Introduction

Myelolipoma is a relatively uncommon, benign mesenchymal tumor composed of mature adipose tissue admixed with benign mature hematopoietic elements in varying proportions. The most common site of involvement is the adrenal gland. The occurrence in extra-adrenal sites is quite rare with an incidence of 0.4% at autopsy [1]. Extra-adrenal myelolipomas may occur in the retroperitoneum, pelvis, presacral area, thorax, mediastinum, stomach, liver and even in the thyroid glands [2]; they are seldom seen in the kidney. Only six cases of myelolipomas arising in the perirenal tissue have been reported [3], with only two previous reports of a renal parenchyma location [2,4]. Here, we present a third case of renal myelolipoma. Radiological and clinicopathologic features are described and differential diagnosis is discussed.

## Case presentation

**Figure 1:**
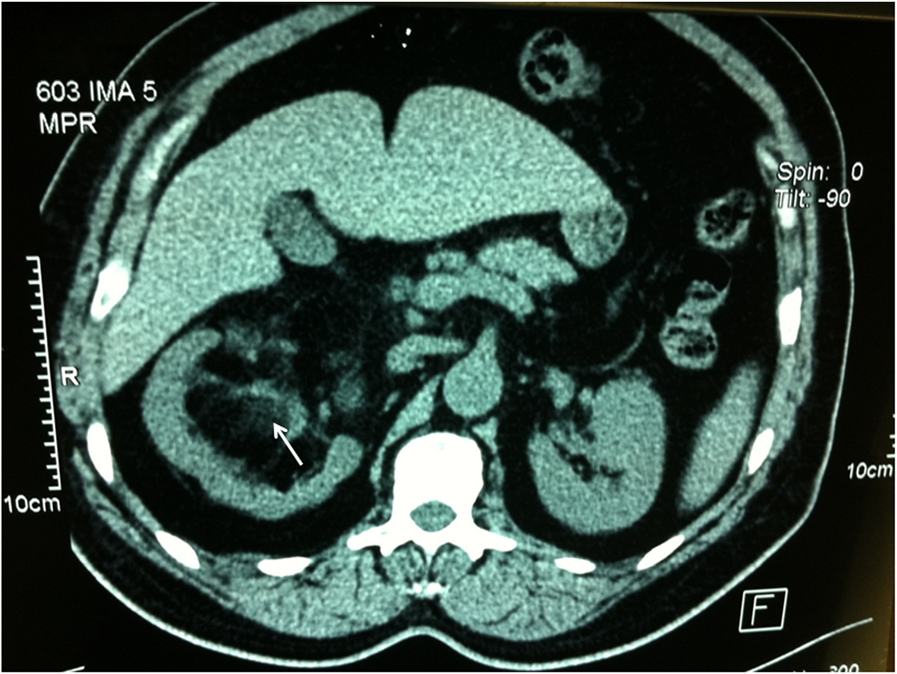
Computed tomography of the abdomen and pelvis showing parenchymal mass of fat density of the right kidney (arrow).

**Figure 2:**
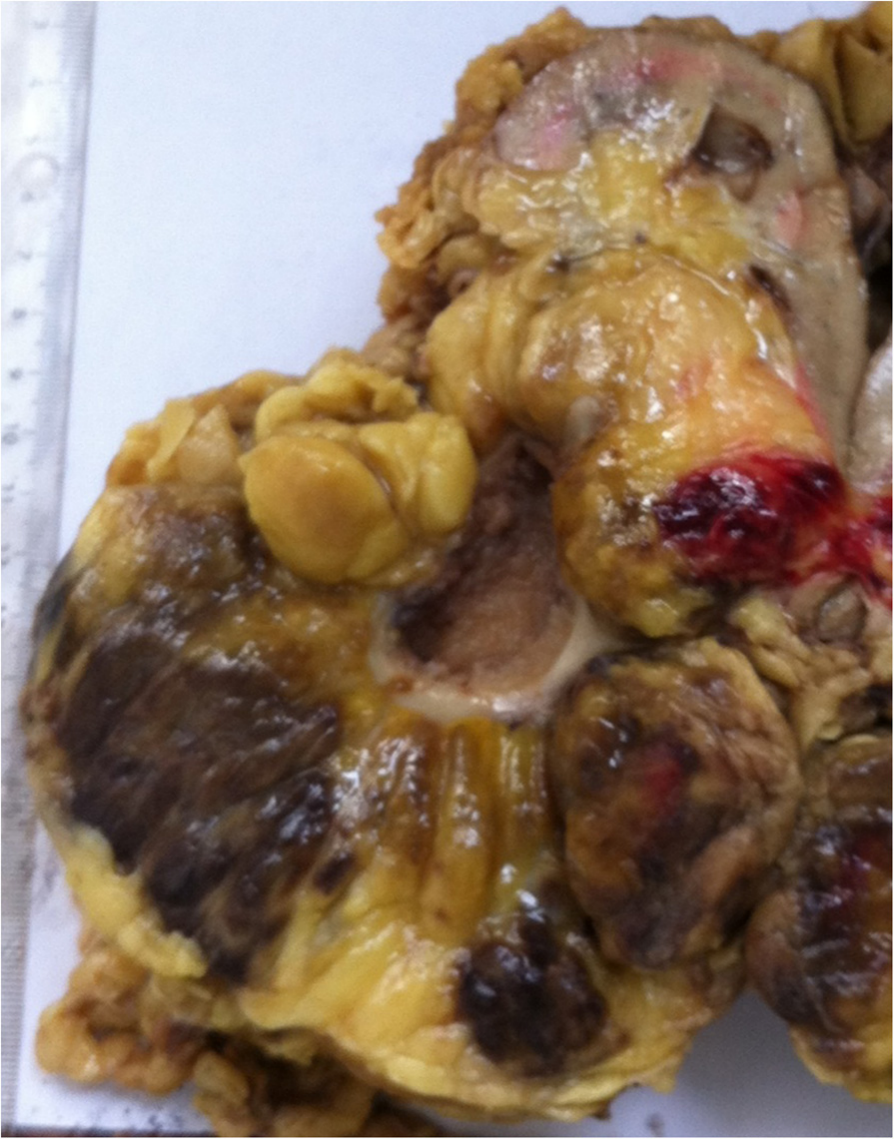
**Gross photogram of the well-circumscribed solid tumor of the right kidney (coronal slice).** The cut surface shows areas of soft yellow fatty tissue admixed with areas of red-brown friable tissue.

**Figure 3:**
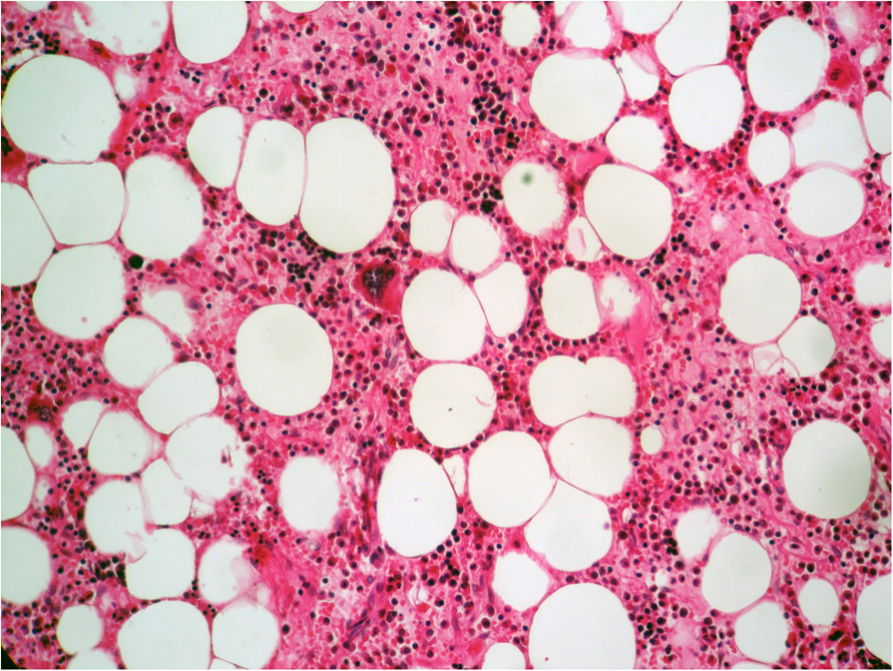
**Histologic findings (hematoxylin and eosin) of the kidney mass.** The tumor is composed of mature adipose tissue and islands of normal hematopoietic elements with intermingled megakaryocytes (original magnification ×100).

**Figure 4:**
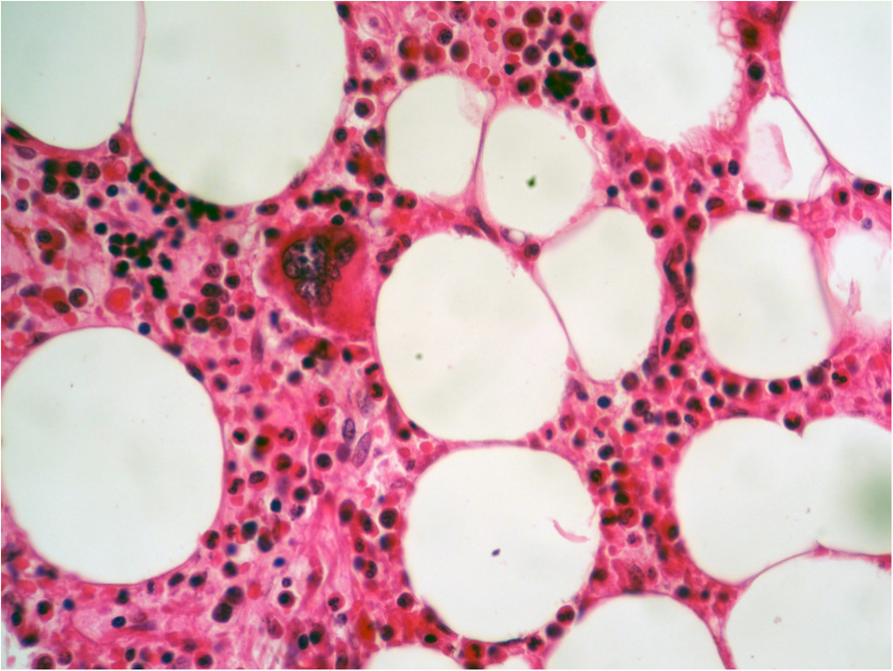
Higher power magnification (×200) showing normal hematopoietic elements, including megakaryocytic, erythropoietic and granulopoietic cell lineages interspersed within adipose tissue.

A 55-year-old Moroccan man, with insulin-dependent type II diabetes, presented with right flank pain. A physical examination did not reveal any other abnormalities, such as hepatosplenomegaly or lymphadenopathy. Laboratory tests (blood count, hemoglobin, uremia, and creatinine clearance) revealed no abnormal findings. Ultrasonography (US) of his right kidney showed hydronephrosis. The patient underwent a left retrograde pyelography which confirmed hydronephrosis caused by calyceal and pelvic lithiasis and revealed a non-functional right kidney. Computed tomography (CT) of his abdomen and pelvis showed a relatively well-circumscribed parenchymal mass of fat density, measuring 10cm in diameter, with no involvement of the perinephric adipose (Figure 1). His adrenal glands were normal and no lymphadenopathy was detected. A laparotomy was performed and the hydronephrotic kidney was removed. On gross examination, the right kidney removed from the patient was irregular in shape, weighed 800g and measured 15×11×8cm. On the lower pole of the kidney, there was a relatively well-circumscribed, non-encapsulated, solid mass, measuring 11×8×8cm. The cut surface showed areas of soft yellow fatty tissue admixed with irregular areas of brownish friable tissue (Figure 2). Histologically, the tumor was composed of mature adipose tissue and nests of hematopoietic precursor cells similar to those found in normal bone marrow (Figure 3). Representation of all the three hematopoietic cell lineages (granulocytic, erythroid and megakaryocytic) was observed (Figure 4). Scattered small lymphoid aggregates and foci of hemorrhage were noted. No adrenal rests were found. Based on this histomorphology, a hematological investigation including bone marrow aspiration was performed to rule out any underlying hematological disorders. Bone marrow aspiration was normocellular. Based on these findings, renal myelolipoma was diagnosed. The patient had an uneventful postoperative course and has remained disease free at 3-month follow up.

## Discussion

Myelolipoma is a relatively uncommon benign tumor, composed of mature adipose tissue and hematopoietic elements. Myelolipoma is most frequently found in the adrenal gland, but it may occur in the pelvis, retroperitoneal space or even in the chest. Extra-adrenal myelolipomas occur in women more often than in men and more often in middle-aged to older patients [5].

The exact pathogenesis of myelolipoma is not clear at present. Most theories involve the development and differentiation of either ectopic adrenal rests or hematopoietic stem cell rests in response to a triggering stimulus, particularly endocrine dysfunction [6]. Other researchers found clonal cytogenetic abnormalities, which suggested it was of tumor origin [7]. Chromosomal translocations (3;21) (q25;p11) detected in myelolipomas and in benign lipomatous neoplasia seen in patients with acute myelogenous leukemia or myelodysplastic syndrome suggest the origin of this tumor to be bone marrow, and may indicate that myelolipoma is derived from erroneously transferred erythroid cells [8].

Both adrenal and extra-adrenal myelolipomas are typically asymptomatic. They tend to be incidentally discovered during radiological investigation of symptoms unrelated to myelolipoma. However, some patients with myelolipoma complain of abdominal or flank pain, possibly related to intratumoral or peritumoral hemorrhage, tumor infarction or mechanical compression from tumor bulk [9]. Because these tumors are rare, criteria for diagnosing extra-adrenal myelolipoma radiologically do not exist. Characteristics of adrenal myelolipoma are extrapolated to apply to these structures which present in a variety of locations [10]. Myelolipoma can be reliably diagnosed by either CT or magnetic resonance imaging (MRI). Radiological imaging typically reveals a well-circumscribed mass with a heterogeneous appearance due to the varying proportions of fat within the mass. Adipose tissue is characterized by low attenuation on CT imaging (i.e., –25 to −100 Hounsfield units). On MRI, fat displays high signal intensity on T1-weighted images whereas the myeloid component of these tumors has a T2-weighted signal. Contrast enhancement with CT scan or MRI will vary depending on the composition of the mass. Soft tissue components enhance whereas adipose tissue does not [11]. Calcification is present in a minority of cases on CT. Because of their characteristic appearance on CT, adrenal myelolipomas can usually be diagnosed without intervention and followed radiographically. Extra-adrenal myelolipomas, however, are more difficult to diagnose preoperatively because they are easily confused with several malignancies. A fatty retroperitoneal mass could essentially be a retroperitoneal liposarcoma, a renal or adrenal myelolipoma, a renal angiomyolipoma or a retroperitoneal teratoma.

If a definite diagnosis is needed, a fine-needle biopsy is indicated either under US or CT guidance, although one should consider the possibility of a ruptured mass or the development of hemorrhage. The biopsy can show adipose tissue and a variable amount of hematopoietic elements, raising the differential diagnosis of extramedullary hematopoiesis and extra-adrenal myelolipoma. It must be noted that it is also often difficult to label a retroperitoneal neoplasm benign based on biopsy, because both benign and malignant tumors can share similar characteristics.

Grossly, extra-adrenal myelolipoma is a solitary circumscribed mass ranging in size from a few centimeters to 27cm [12]. The tumor is usually spherical to ovoid, well circumscribed, sometimes surrounded by a pseudocapsule. The cut surface typically has a variegated appearance, with areas of greasy-appearing soft yellow tissue alternating with irregular areas of dark red-brown friable tissue. Microscopically, the tumor is composed of a variable admixture of mature adipose tissue with islands and nests of hematopoietic elements of different percentages. The cellularity of hematopoietic precursors is variable and the three hematopoietic cell lineages (granulopoietic, erythropoietic and megakaryocytic) are present. In some cases, areas of infarction, hemorrhage, and rarely foci of calcification are noted [9]. Immunohistochemical staining and molecular testing are of no clinical or histological benefit.

When the diagnosis of retroperitoneal myelolipoma is considered, it should be differentiated from other fat-containing retroperitoneal tumors, of which liposarcoma is the most common. Liposarcoma involves middle-aged adults with a peak incidence in the 6^th^ decade, with no sex predominance. Retroperitoneal liposarcomas are often asymptomatic until the tumor has exceeded 20cm in diameter and may be found incidentally. In renal localization, liposarcomas are rare with few cases reported in the literature [13]. Macroscopically, a liposarcoma consists usually of a large, well-circumscribed, lobulated mass. Color varies from yellow to white depending on the proportion of adipocytic, fibrous and/or myxoid areas. Areas of necrosis are common in large lesions. Histologically, a liposarcoma is composed either entirely or in part of a mature adipocytic proliferation showing a significant varying number of lipoblasts. In our case, the tumor contained, in addition to mature adipose tissue, nests of hematopoietic elements, and no histological sign of malignancy was found.

Angiomyolipoma is a benign mesenchymal tumor usually occurring in the kidney. It comprises 2.0% to 6.4% of all renal tumors; however, it represents one of the most common benign renal lesions [14]. It can occur sporadically or in patients with tuberous sclerosis. Histologically, angiomyolipoma is a triphasic tumor composed of varying amounts of thick-walled dysplastic or dysmorphic blood vessels, spindle and epithelioid smooth muscle cells and mature adipose elements. Although the diagnosis of angiomyolipoma is usually straightforward, some cases showing predominance of any one of the angiomyolipoma components may mimic a number of lesions, including myelolipoma and liposarcoma (fat-predominant angiomyolipoma) [14].

Also considered in the differential diagnosis are reactive extramedullary hematopoietic ‘tumors’ that usually occur in the context of myeloproliferative disorders or chronic hemolytic anemia [5]. Both renal extramedullary hematopoietic tumors and myelolipomas are rare. Although the components of these tumors are the same, we consider them separable clinically, pathogenetically and, in many cases, pathologically. Patients with extramedullary hematopoietic tumors are characterized by anemia, frequent hepatosplenomegaly, and abnormal peripheral blood smears, and may be any age. The tumors are usually multiple and usually located in the mediastinum or epidural space. By contrast, patients with extra-adrenal myelolipomas are usually older than 40 years, have normal blood studies, absent hepatosplenomegaly, and usually have chronic debilitating diseases or endocrinopathies. Extra-adrenal myelolipomas are single and usually occur in the abdomen. Clinical and biological examinations of our patient with diabetes showed no hepatosplenomegaly and no underlying hematological abnormalities. A CT scan showed a single renal mass. In contrast to extra-adrenal myelolipomas that are well circumscribed, extramedullary hematopoietic tumors lack circumscription and are ill defined. Microscopically, extramedullary hematopoietic tumors have a predominance of hematopoietic elements, with erythroid hyperplasia. Fat is not an enlarged component of the process [5]. Extra-adrenal myelolipomas may have a predominance of either the hematopoietic or fatty component, chiefly the latter, and generally have a more conspicuous lymphocyte population [15]. The presence of megakaryocytes is considered to be essential for the diagnosis of extra-adrenal myelolipoma [5]. In our case, the tumor was well-circumscribed and, histologically, the fat component was predominant. The hematopoietic component was represented by nests of hematopoietic elements, including numerous megakaryocytes, accompanied with foci of lymphoid cells. Extra-adrenal myelolipoma is also distinct from true bone marrow in that no reticular sinusoids or bone spicules are present. However, extra-adrenal myelolipomas containing bone spicules have been reported [16]. The bone spicules are thought to be the result of osseous metaplasia.

Retroperitoneal myelolipomas should also be differentiated from other lesions that contain adipose tissue and hematopoietic elements, such as teratomas. Histology of the latter shows tissue elements from all the three germ layers.

The usual natural history of extra-adrenal myelolipoma is benign although they may enlarge and bleed. Some reports have shown stable lesions with follow up from 3 to 62 months [16]. Small asymptomatic lesions may be managed expectantly. Radiological follow up is recommended due to the potential for growth and hemorrhage. In patients who are symptomatic, have an unclear diagnosis or possess an enlarging tumor mass, as in our patient, surgical intervention is warranted. Long-term prognosis is excellent.

## Conclusion

In conclusion, our case is noteworthy because the tumor location was extremely rare. Despite its rarity, renal myelolipoma should be known and mentioned in case of adipose-containing tumor. Management options for extra-adrenal myelolipoma include conservative and surgical approaches depending upon the certainty of the diagnosis and the progression of the patient’s symptoms. It is generally impossible to distinguish this entity from other retroperitoneal tumors by radiographic differentiation. So the final diagnosis should depend on pathological features of the surgically removed sample.

## Consent

Written informed consent was obtained from the patient for publication of this case report and accompanying images. A copy of the written consent is available for review by the Editor-in-Chief of this journal.

## Abbreviations

CT: Computed tomography; MRI: Magnetic resonance imaging; US: Ultrasonography.

## Competing interests

The authors declare that they have no competing interests.

## Authors’ contributions

MG retrieved clinical information, wrote the manuscript and performed the literature review. KZ first identified this case, proposed the study and revised the manuscript for important intellectual content. AJ acquired photomicrographs. FZ and ZB provided valuable insight during manuscript preparation. NM supervised the entire case. All authors read and approved the final manuscript.
